# Homozygous CALR Mutation in Primary Myelofibrosis and Its Effect on Disease Phenotype: A Case Report and Review of the Literature

**DOI:** 10.1155/2019/1430170

**Published:** 2019-01-20

**Authors:** Qurratulain Rizvi, Uzma Zaidi, Saba Shahid, Shariq Ahmed, Tahir Shamsi

**Affiliations:** ^1^Department of Clinical Haematology, National Institute of Blood Diseases & Bone Marrow Transplantation, P.O. Box-75300, Karachi, Pakistan; ^2^Department of Genomics, National Institute of Blood Diseases & Bone Marrow Transplantation, P.O. Box-75300, Karachi, Pakistan

## Abstract

Somatic mutations in *CALR* gene have been reported in 60%–88% of patients with essential thrombocythemia (ET) and primary myelofibrosis (PMF) who are negative for *JAK2* and *MPL* mutations. Most of the *CALR* mutations analyzed to date are heterozygous mutations in exon 9 of the gene. Homozygosity in *CALR* gene is rarely reported, and its association with clinical behavior of disease and impact on outcome of patients is not studied so far. We herein report a case of intermediate-2 risk PMF (according to IPSS) diagnosed with homozygous mutation (c.1139delA p.E380fs^*∗*^50) in *CALR* gene having severe disease manifestations at presentation.

## 1. Introduction

Primary myelofibrosis (PMF) is a classical Philadelphia-negative myeloproliferative neoplasm (MPN) characterized by abnormal proliferation of predominantly megakaryocytes and granulocytes. The disease involves remodeling of bone marrow, including progressive myelofibrosis, exaggerated angiogenesis, and extramedullary hematopoiesis manifested by leukoerythroblastic blood smear and hepatosplenomegaly [1]. A number of molecular pathways and mutations play role in the pathogenesis of the disease making it a complex disorder.

Approximately, 50% of patients with PMF carry the *JAK2*‐V617F mutation, whereas mutations of *MPL* are found in an additional 5%. Somatic mutations in *CALR* gene were detected in 70% to 84% of *JAK2* nonmutated MPN [2]. An entity defined as triple-negative primary myelofibrosis with absent *JAK2*, *CALR*, and *cMPL* mutation is identified, being associated with reduced overall survival [3]. Most of the *CALR* mutations analyzed to date are heterozygous mutations in exon 9 of the gene. Patients with heterozygous *CALR*-mutant PMF have distinct clinical features, an indolent clinical course, and better survival compared with PMF patients carrying *JAK2* (V617F) or an *MPL* exon 10 mutation [4, 5]. Due to the high prevalence of *CALR* mutation in PMF, WHO has incorporated its presence in the revised diagnostic criteria of prefibrotic and overt myelofibrosis [6]. Homozygosity in *CALR* gene is rarely reported, and its association with clinical behavior of disease and impact on outcome of patients is not studied so far [7]. We herein report a case of PMF diagnosed with homozygous mutation in *CALR* gene having severe disease manifestations at presentation.

## 2. Case Scenario

**Figure 1:**
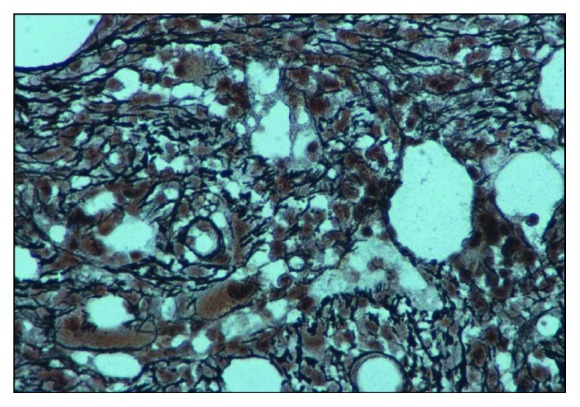
Reticulin stain showing MF grade 2 fibrosis.

**Figure 2:**
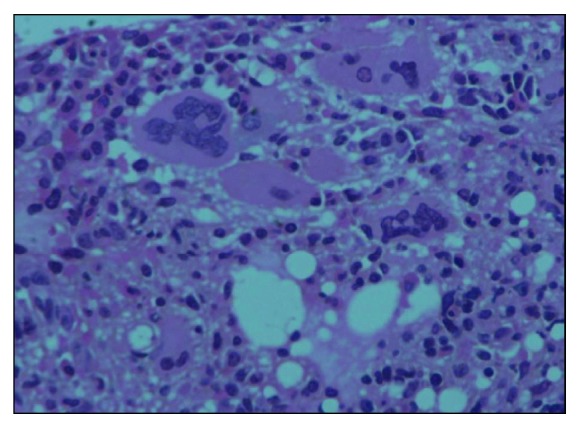
Trephine biopsy showing atypical megakaryocytes.

A 57-year-old gentleman, known diabetic, presented with complaints of easy fatigability, loss of appetite, low-grade fever, and left hypochondrial discomfort for the past 2 months. Physical examination revealed splenomegaly (8 fingers below the left costal margin). Complete blood count showed Hb of 9.4 g/dl, TLC of 5.8 × 10^9^/L, and Plt count of 337 × 10^9^/L with leukoerythroblastic picture and absent circulating blasts. Bone marrow findings were consistent with primary myelofibrosis, exhibiting hypercellularity with marked increase in reticulin fibrosis (MF grade 2, according to EUMNET consensus, Figure 1) and atypical, hypolobated megakaryocytes forming loose clusters (Figure 2). Blast percentage was less than 5%. Cytogenetic analysis showed normal male karyotype. BCR-ABL translocation via PCR, *JAK2*‐V617F, and *MPL* mutation were not detected. Associated *CALR* mutations were not determined via next-generation sequencing due to resource constraints. Serum erythropoietin level was 200 U/L. According to the International Prognostic Scoring System (IPSS), the patient was falling into intermediate-2 risk category. He was initially prescribed ruxolitinib 10 mg twice daily (gradually escalated to 15 mg twice daily) along with erythropoietin 20,000 IU weekly for correction of anemia.

## 3. Methodology

The research protocol was approved by the Institutional Review Board (ERC/IRB) and conformed to the tenets of the Declaration of Helsinki. Written informed consent was obtained from the patient. The diagnosis was established according to the 2016 WHO criteria, and the clinical and laboratory data were reviewed from medical record.

### 3.1. Mutation Analysis

The DNA was extracted from the peripheral blood sample of the MPN patient using a QIAamp DNA Mini Kit (Qiagen, Hilden, Germany) according to the manufacturer's instructions. The *JAK2* and *MPL* mutations were analyzed by using the ARMS PCR for *JAK2*‐V617F and *MPL* W515L/K mutations as previously described [8]. Sanger sequencing was performed to screen *CALR* gene for exon 9. The genomic region of interest was amplified by PCR. Amplification of *CALR* exon 9 was performed by using previously reported primers *CALR*. –F (5′-ACAACTTCCTCATCACCAACG-3′) and *CALR*–R (5′- GGCCTCAGTCCAGCCCTG -3′) [8]. The total reaction volume of 20 *μ*l contained approximately 50 ng of DNA, 200 nmol/l for forward and reverse primers, deoxynucleotide triphosphates (dNTPs, 200 *µ*mol/l each), 1 unit of Dream Taq DNA Polymerase (Thermo Scientific, Life Technologies Inc, Carlsbad, California 92008). Samples were amplified using the following PCR conditions: 94°C for 10 minutes; 30 cycles of 94°C for 30 seconds; 59°C for 30 seconds; 72°C for 30 seconds with final extension of 72°C for 10 minutes and hold at 4°C. PCR products were analyzed using 2% agarose gel electrophoresis. Sequencing products were purified, and the sequencing reaction was performed using the BigDye terminator cycle sequencing kit, v3.1 (Applied Biosystems®, California, USA) and was analyzed on an automated DNA analyzer (ABI, 3500).

## 4. Results and Discussion

**Figure 3:**
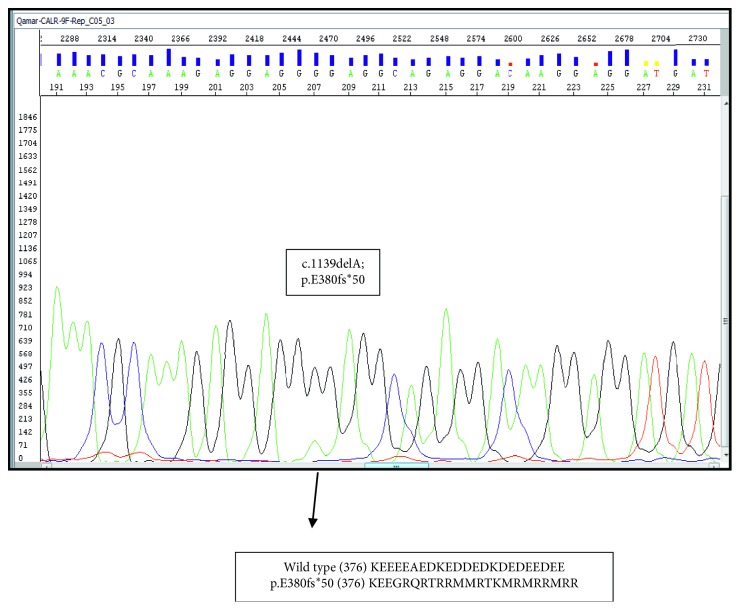
Electropherogram of *CALR* exon 9 homozygous mutation (c.1139delA; p.E380fs^*∗*^50) and the new peptide sequence.

Sanger sequencing for *CALR* exon 9 mutation identified a homozygous mutation (c.1139delA p.E380fs^*∗*^50) in this case as shown in Figure 3. The same mutation has been reported in Chinese population in heterozygous state which disturbed the reading frame shift and significantly altered C-terminal domain of *CALR* protein [9]. This is a unique finding, as homozygosity in *CALR* gene has been exclusively associated with type 2 mutation (5 bp insertion; 1). Basically, *CALR* mutations do not alter the primary structure of the *CALR* binding site for glycoproteins but affect the C-terminal domain, which contains a KDEL motif and a Ca^2+^ binding domain [10].

The advent of mutation in *CALR* gene changed the landscape of MPNs. It was first recognized as a somatic mutation in patients with MPNs who had no mutations in either *JAK2* or *MPL* by Klampfl et al. in 2013 [7]. Calreticulin is a protein found in endoplasmic reticulum, cytoplasm, or cell surface, which maintains the calcium hemostasis, regulates the cell proliferation, phagocytosis, and apoptosis, and also ensures proper glycoprotein folding [11]. Patients with heterozygous *CALR*-mutated PMF are usually males, younger as compared to *JAK2*-mutated cases, and they have myeloproliferation more specific to the megakaryocytic lineage, thus presenting with a more pronounced thrombocytosis. They usually have low hemoglobin and white cell counts. There is low incidence of thrombotic complications and longer survival reported in this group of patients. This prognostic impact of *CALR* in PMF is limited to type 1 mutation, with type 2 having similar prognosis as that of *JAK2*-mutated PMF [12].

Mutated *CALR* homozygosity seems to be a rare event in MPNs and is reported with type 2 mutations in exon 9. In the study by Klampfl et al., three of 289 *CALR*-mutated patients were homozygous for the mutation, and all had type 2 mutations [7]. Similar frequencies of acquired 19pUPD for both *CALR* insertions and deletions were found by Nangalia et al. [13]. There is not much literature to provide specific evidence concerning homozygous *CALR* mutations and its impact on the phenotypic behavior of the disease as well as on the type of *CALR* mutation.

Homozygous *CALR* mutation in PMF has been associated with acquired myeloperoxidase (MPO) deficiency as reported by Alexandre et al. [14]. An individual case report of homozygous *CALR* type 1 mutation has been described in a patient with an atypical BCR-ABL1-positive MPN [15].

Our index case was diagnosed in the fibrotic phase of PMF. He was found to be in intermediate-2 risk category according to IPSS. His molecular mutation profile was negative for *JAK2*, *MPL*, and BCR-ABL translocation. Interestingly, the mutation in *CALR* gene detected in this patient was homozygous mutation (c.1139delA p.E380fs^*∗*^50), which seems to be a type 2-like mutation based on the classification according to the absence of (KDEL) motif in the wild type and modification in the alpha helix structure. These mutations produce a new C-terminus which results in loss of endoplasm reticulum (ER) retention signals but maintain the structural integrity of protein. The resultant mutant *CALR* was found in ER without accumulation on the cell surface or Golgi apparatus [13]. This structural modification affects the function and leads to decrease in Ca^+2^ export from the ER and causes downregulation of the calreticulin-NFAT signaling pathway which is directed more towards myeloid lineage commitment [16].

In contrast to the clinical findings associated with heterozygous mutations, i.e., lesser disease severity, thrombocytosis at baseline, and overall good prognosis, our patient with homozygous mutation presented in the advanced phase of disease with constitutional symptoms including weight loss, anorexia, fatigue and low-grade fever, and massive splenomegaly requiring treatment. His platelet count was within normal range at presentation. He was offered *JAK2* inhibitor and erythropoiesis-stimulating agent. After a period of 6 months, the patient was reevaluated using dynamic IPSS (DIPSS) and fell into the intermediate-2 risk category. No thrombotic or hemorrhagic event was noted during the follow-up period. There was mild improvement in constitutional symptoms with ruxolitinib, but red cell transfusion requirement gradually increased. Though initial therapy with ruxolitinib is associated with drop in hemoglobin levels, the severe disease manifestations at presentation compels us to consider that homozygous mutations in *CALR* gene might be accountable for an aggressive disease phenotype in PMF.

## 5. Conclusion

As *CALR* mutation in MPNs is one of the recent advances, its extensive impact on baseline characteristics and clinical behavior of patient, disease outcome, and risks and benefits in long term is yet to be explored. Prospective studies are needed to elucidate the influence of *CALR* mutation in MPNs with extensive focus on the homozygous pattern of mutation.
